# Life history of *Mecideamajor* with descriptions of nymphal instars (Hemiptera, Heteroptera, Pentatomidae)

**DOI:** 10.3897/zookeys.796.21325

**Published:** 2018-11-15

**Authors:** C. Scott Bundy, J. E. McPherson

**Affiliations:** 1 Department of Entomology, Plant Pathology, and Weed Science, New Mexico State University, Las Cruces, NM 88003, USA New Mexico State University Las Cruces United States of America; 2 Department of Zoology, Southern Illinois University, Carbondale, IL 62901, USA Southern Illinois University Carbondale United States of America

**Keywords:** description, eggs, life history, *
Mecidea major
*, Pentatomidae

## Abstract

The life history of the stink bug *Mecideamajor* Sailer was studied in the southern half of New Mexico primarily from January 2005 through December 2007, and the nymphal instars were described. This species was active throughout the year as adults and nymphs, including the winter months. It occurred on various species of grasses during this time but primarily on Lehmann lovegrass, *Eragrostislehmanniana* Nees; grama grasses, *Bouteloua* spp.; and tobosagrass, *Pleuraphismutica* Buckley. The times of occurrence and abundance of the eggs, nymphs, and adults suggest this species is bivoltine with the possibility of a third generation. Instars can be distinguished by several morphological features including body size and presence and relative development of wing pads.

## Introduction

The stink bug genus *Mecidea* Dallas (Pentatomidae: Pentatominae: Mecideini), which apparently is associated with xeric and semixeric environments, occurs within the subtropical and adjacent temperate regions of the world (Sailer 1952). This phytophagous genus, which contains 17 species (Sailer 1952, Schuh and Slater 1995), is represented in America north of Mexico by three species: two species, *M.major* Sailer and *M.minor* Ruckes, collectively, occur from the Midwest to California (Sailer 1952, Froeschner 1988, McPherson et al. 2009); the third species, *M.longula* Stål, recently has been found in south Florida (Eger and Dobbs 2010).

*Mecideamajor* ranges from Minnesota (Koch et al. 2014), southern Illinois (McPherson and Vogt 1981), and Missouri west to Arizona (Sailer 1952), and south to Mexico (Thomas 2000). It also has been recorded from Florida (Mead 1988), but Eger and Dobbs (2010) speculated that it probably was an adventitious specimen, perhaps blown in by a hurricane. Little is known about its biology, including its nymphal stages. However, it has been reared in the laboratory (Bundy et al. 2005) and the egg described (Bundy and McPherson 2005).

*Mecideamajor* commonly is found from July to October (Sailer 1952) but has been collected in every month of the year (Sailer 1952, Jones 1993). It apparently is a grass specialist but has been found on both grass and nongrass species (Sailer 1952, Bundy 2004). Host plants include side-oats grama, *Boutelouacurtipendula* (Michaux); sorghum, *Sorghumhalapense* (L.); wheat, *Triticumaestivum* L.; “grasses;” spinach, *Spinaciaoleracea* L.; cotton, *Gossypiumhirsutum* L.; *Senecio* (Sailer 1952); wild oat, *Avenafatua* L.; Bermuda grass, *Cynodondactylon* (L.); barnyard grass, *Echinochloacrusgalli* (L.); bush muhly, *Muhlenbergiaporteri* Beal; *Bromus* sp. (Jones 1993); common oat, *Avenasativa* L.; black grama, *Boutelouaeriopoda* (Torrey); blue grama, *Boutelouagracilis* Humboldt, Bonpland, & Kunth; common barley, *Hordeumvulgare* L.; foxtail barley, *Hordeumjubatum* L.; mesa dropseed, *Sporobolusflexuosus* (Thurber ex Vasey) Rydberg (Bundy 2004); Wright’s threeawn, *Aristidapurpurea* Nuttall; tobosagrass, *Pleuraphismutica* Buckley (Bundy 2004, Bundy and McPherson 2005); Lehmann lovegrass, *Eragrostislehmanniana* Nees (Jones 1993, Bundy et al 2005); *Baccharisneglecta* Britton (Palmer 1987); guayule, *Partheniumargentatum* Gray (Stone and Fries 1986); and threadleaf snakeweed, *Gutierreziamicrocephala* (DC.) A. Gray (as *Zanthocephalummicrocephala*) (Foster et al. 1981).

Scattered notes have been published on the field life cycle of *M.major*. Jones (1993) collected second to fifth instars in Arizona on several grass species (e.g., Bermuda grass, Lehmann lovegrass) from early March to early June. He also reported that females caged on potted Lehmann lovegrass (no date given) deposited eggs in two rows of 12–14 at the bases of the stems near the surface of the soil.

During 2003 and 2004 several reproducing populations of *M.major* were found in the southern half of New Mexico on various species of range grasses, primarily on Lehmann lovegrass, *Eragrostislehmanniana*; grama grasses, *Bouteloua* spp.; and tobosagrass, *Pleuraphismutica*. The number of bugs and presence of various instars suggested that the populations were large enough for a life history study including descriptions of the instars. Presented here are the results of that study.

## Materials and methods

### Life history

The field study was conducted in Las Cruces (Doña Ana Co.), New Mexico, from January 2005 through December 2007, supplemented with additional observations in spring and summer of 2017. Two field sites (site 1: 32°20'55.5"N, 106°44'28.8"W, altitude 1202 m; site 2: 32°19'35.2"N, 106°45'10.4"W, altitude 1252 m) were selected: vegetation at site 1 was predominately Lehmann lovegrass with scattered populations of tobosagrass and grama grasses; that at site 2 was Lehmann lovegrass and Bermuda grass.

Weekly sampling was initiated at both sites in late January 2005 and continued through mid-December 2007. Approximately six sets of 25 sweeps were taken with a sweep net (38 cm diam.) at each site per date, counts of nymphs and adults recorded when possible, and the animals released. Specimens that could not be determined to instar(s) were preserved in 80% ethanol (EtOH) for closer examination in the laboratory. Eggs were recorded from visual observations. Representative samples of the eggs and nymphs were preserved in 80% EtOH and examined in the laboratory to spot-check field determinations. During each collecting trip, observations were made on the bugs’ activities and development on host plants. Life history data on reproduction and development for the 3 years of this study, plus the observational data from 2017 noted above, were combined to gain a better understanding of the annual life cycle.

### Descriptions of immature stages

The egg previously has been described by Bundy and McPherson (2005). The description of each instar is based on ten field-collected individuals. Nymphs were selected from field samples preserved in 80% EtOH that had been collected earlier for spot-checking of instars. Drawings were made on a light box using digital photographs taken through a dissecting microscope. Measurements (in millimeters) were made with an ocular micrometer.

### Statistics

Measurements are expressed as means ± SE; standard errors <0.005 are listed as 0.00.

### Voucher specimens

Samples of instars have been vouchered in the New Mexico State Arthropod Collection in Las Cruces, NM.

## Results and discussion

### Life history

**Figure 1.**
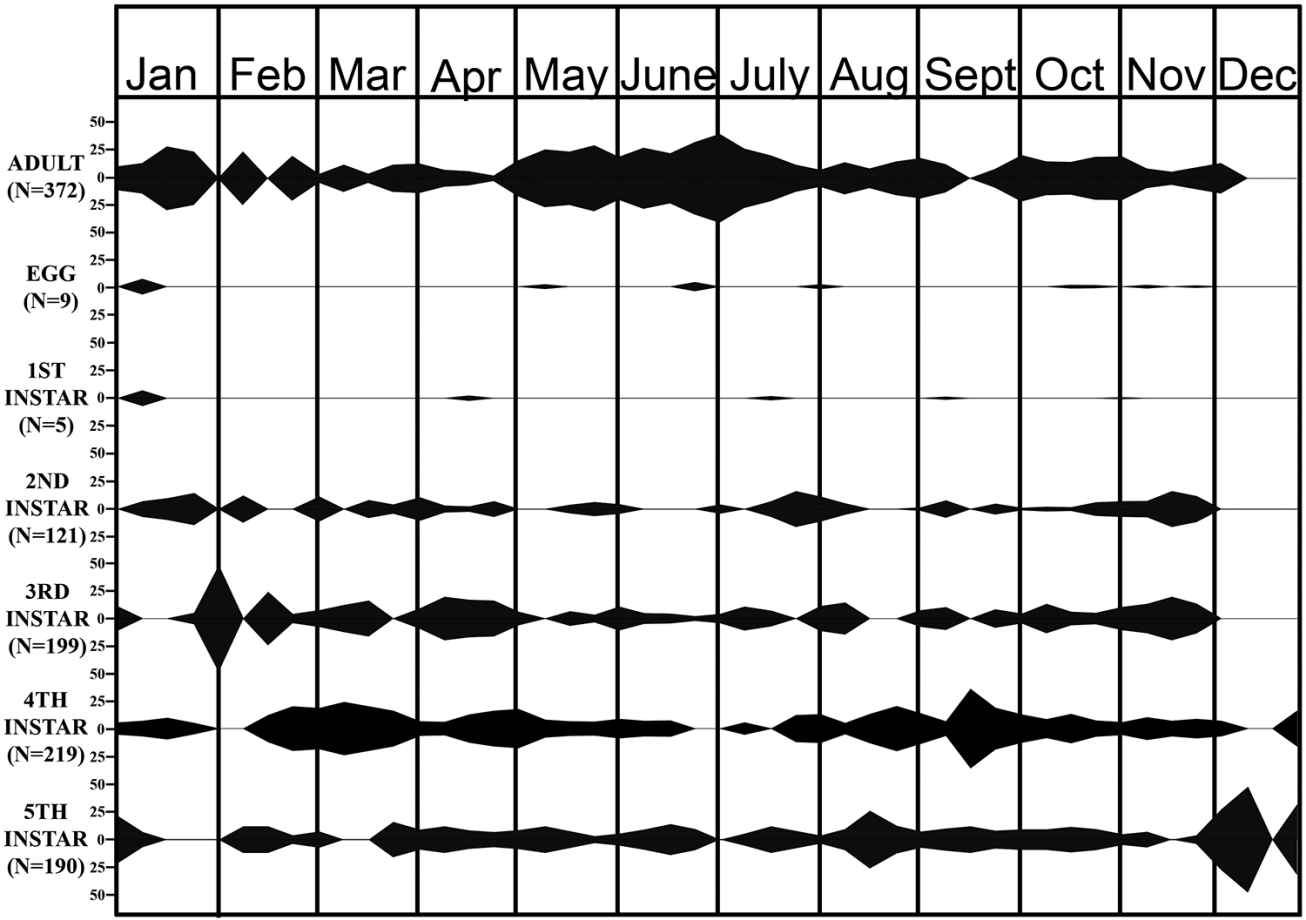
Field life cycle of *M.major*. Percentage in each sample of individuals of each stage collected during 2005-2007 in Las Cruces, NM. Beginning and end points of each shaded area represent sample dates preceding and following collections of specimens, respectively.

**Figure 2.**
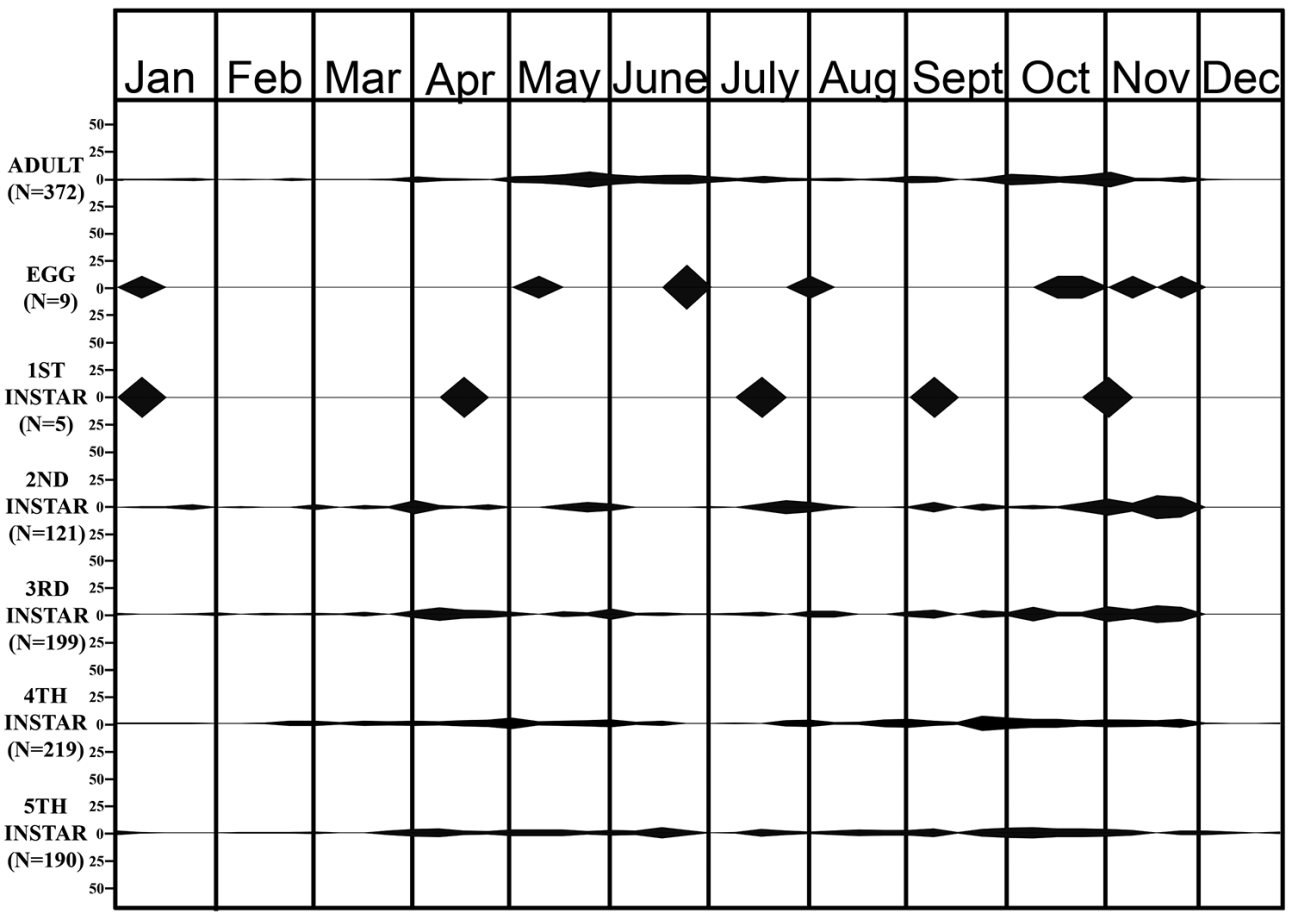
Field life cycle of *M.major*. Percentage in each sample of total individuals of same stage collected during 2005-2007 in Las Cruces, NM. Beginning and end points of each shaded area represent sample dates preceding and following collections of specimens, respectively.

This species was active through the year, including the winter months (Figs 1, 2). Adults, eggs, and all instars were collected in October and November but only adults, fourths, and fifths in December. However, all stages, including eggs, were found in January. This strongly suggests that only older individuals (adults, 4ths, and 5ths) successfully overwintered and produced the younger instars (1sts–3rds) found in January.

Eggs (*n* = 86, nine clusters, ≈10 eggs per cluster [9.6 ± 3.19; range 3–14]) were collected sporadically from early January to late November (Figs 1, 2). They were laid in regular alternating double rows of 3 to 14 eggs, on the undersides of leaves, flowers, and maturing heads of *E.lehmanniana*. As with many other pentatomids, eggs were glued to one another and to the substrate. They were yellowish white when deposited, cream-colored after 1–3 days, and light brown at maturity. Eyespots were visible in 4–5 days. The egg burster was visible within 2–3 days of hatching (Bundy and McPherson 2005).

The first instars (a non-feeding stage) were collected in January, April, July, September, and October (*n* = 5); the sample size was low because first instars are small and tend to cluster. The second-fifth instars were collected almost continuously from January into November (second and third instars) and December (fourth and fifth instars) (Figs 1, 2). All five instars were found on *E.lehmanniana*, *P.mutica*, *C.dactylon*, and *Bouteloua* spp.

The number of generations per year in this species is difficult to determine because of the marked overlap of the times of occurrences of the instars and the generations and the lack of definite peaks in abundance during each generation. Based on the early presence of all stages in the field (January), all stages might have overwintered. However, it is more likely that adults, fourth, and fifth instars, which were collected in December, overwintered, whereas eggs and first–third instars began development in January. In either scenario, the result would be spring and summer generations, with the summer generation reaching adults in the fall. If these adults did not reproduce during late fall, this would represent a bivoltine species. However, if adults produced some offspring in the fall that overwintered, this would represent a partial third generation. We believe a partial third is more likely for southern New Mexico. Specimens collected in 2017 supported the life history data from the 2005–2007 study.

### Descriptions of immature stages

**Egg.** See description in Bundy and McPherson (2005) and Bundy et al. (2005).

**Instars.** The first instar is described in detail, but only major changes from previous instars are described for subsequent instars. Length was measured from the apices of the tylus and juga to the apex of abdomen (two measurements), and width across the mesonotum or abdominal segments 3–4, whichever was widest (both measurements shown). Additional measurements are given in Table 1.

**Table 1.** Measurements (means ± SE, mm) of *Mecideamajor* instars^a^.

	Nymph				
	First instar	Second instar	Third instar	Fourth instar	Fifth instar
Body length^b^	1.18±0.03	2.06±0.03	3.17±0.07	4.51±0.15	7.62±0.17
Body length^c^	1.16±0.03	2.02±0.03	3.16±0.06	4.53±0.15	7.78±0.17
Head length^d^	0.49±0.01	0.59±0.01	0.76±0.01	1.05±0.01	1.35±0.01
Head length^e^	0.44±0.01	0.54±0.01	0.74±0.01	1.09±0.01	1.49±0.02
Thorax width^f^	0.68±0.02	0.92±0.02	1.31±0.01	2.00±0.04	2.92±0.06
Abdomen width^g^	0.72±0.03	1.09±0.02	1.75±0.04	2.40±0.09	2.99±0.11
Width across eyes	0.50±0.01	0.66±0.01	0.82±0.01	1.12±0.01	1.45±0.02
Synthlipsis	0.38±0.00	0.48±0.01	0.58±0.01	0.79±0.01	0.97±0.02
Antennal segments					
First	0.10±0.00	0.16±0.00	0.24±0.01	0.33±0.01	0.45±0.01
Second	0.18±0.00	0.42±0.01	0.65±0.01	1.06±0.02	1.65±0.05
Third	0.14±0.00	0.30±0.01	0.44±0.01	0.65±0.01	0.93±0.02
Fourth	0.28±0.01	0.42±0.01	0.50±0.01	0.67±0.01	0.83±0.01
Labial segments					
First	0.13±0.00	0.22±0.00	0.31±0.01	0.44±0.01	0.62±0.02
Second	0.19±0.02	0.39±0.00	0.50±0.01	0.73±0.01	1.02±0.02
Third	0.14±0.00	0.22±0.01	0.30±0.01	0.36±0.01	0.51±0.01
Fourth	0.18±0.00	0.27±0.01	0.32±0.00	0.41±0.01	0.57±0.01
Notal lengths^h^					
Pronotum	0.12±0.00	0.22±0.01	0.34±0.01	0.54±0.01	0.94±0.02
Mesonotum	0.08±0.00	0.16±0.00	0.32±0.00	0.66±0.01	1.33±0.04
Metanotum	0.03±0.00	0.04±0.00	0.05±0.01	0.07±0.00	0.04±0.00
Leg lengths					
Protrochanter	0.12±0.00	0.16±0.01	0.20±0.01	0.30±0.01	0.42±0.01
Profemur	0.26±0.01	0.45±0.01	0.63±0.01	0.99±0.01	1.46±0.04
Protibia	0.31±0.01	0.53±0.02	0.74±0.01	1.10±0.02	1.61±0.03
Protarsus	0.22±0.00	0.28±0.01	0.36±0.01	0.52±0.02	0.75±0.02
Protarsomeres^i^					
First	0.08±0.00	0.11±0.00	0.16±0.01	0.25±0.01	0.39±0.01
Second	0.18±0.00	0.22±0.01	0.26±0.00	0.35±0.01	0.48±0.01
Mesotrochanter	0.12±0.00	0.16±0.01	0.21±0.01	0.27±0.01	0.40±0.02
Mesofemur	0.28±0.01	0.45±0.02	0.59±0.01	0.89±0.01	1.27±0.02
Mesotibia	0.31±0.01	0.53±0.02	0.73±0.01	1.05±0.02	1.53±0.03
Mesotarsus	0.22±0.00	0.29±0.01	0.37±0.01	0.50±0.01	0.70±0.01
Mesotarsomeres^i^					
First	0.08±0.00	0.11±0.00	0.17±0.01	0.23±0.00	0.35±0.01
Second	0.18±0.00	0.22±0.01	0.27±0.00	0.34±0.01	0.44±0.01
Metatrochanter	0.12±0.00	0.15±0.01	0.21±0.01	0.32±0.01	0.44±0.01
Metafemur	0.29±0.01	0.51±0.01	0.92±0.02	1.14±0.03	1.86±0.03
Metatibia	0.37±0.01	0.66±0.02	0.36±0.01	1.44±0.03	2.19±0.04
Metatarsus	0.23±0.00	0.27±0.01	0.36±0.01	0.52±0.01	0.75±0.02
Metatarsomeres^i^					
First	0.08±0.00	0.12±0.00	0.16±0.00	0.24±0.00	0.38±0.01
Second	0.18±0.00	0.22±0.01	0.27±0.00	0.35±0.01	0.47±0.01

^a^Measurements based on 10 individuals per instar. ^b^Measured from apex of tylus to apex of abdomen with head in normal declivent position. ^c^Measured from apex to juga (often exceeding tylus) to apex of abdomen with head in normal declivent position. ^d^Measured from apex of tylus to apex of head in horizontal position. ^e^Measured from apex of juga to apex of head in horizontal position. ^f^Measured across mesonotum. ^gf^Measured across abdominal segments 3–4 . ^h^Measured across midline. ^i^Total length of measured segments > overall length because of curvature.

**Figures 3–5.**
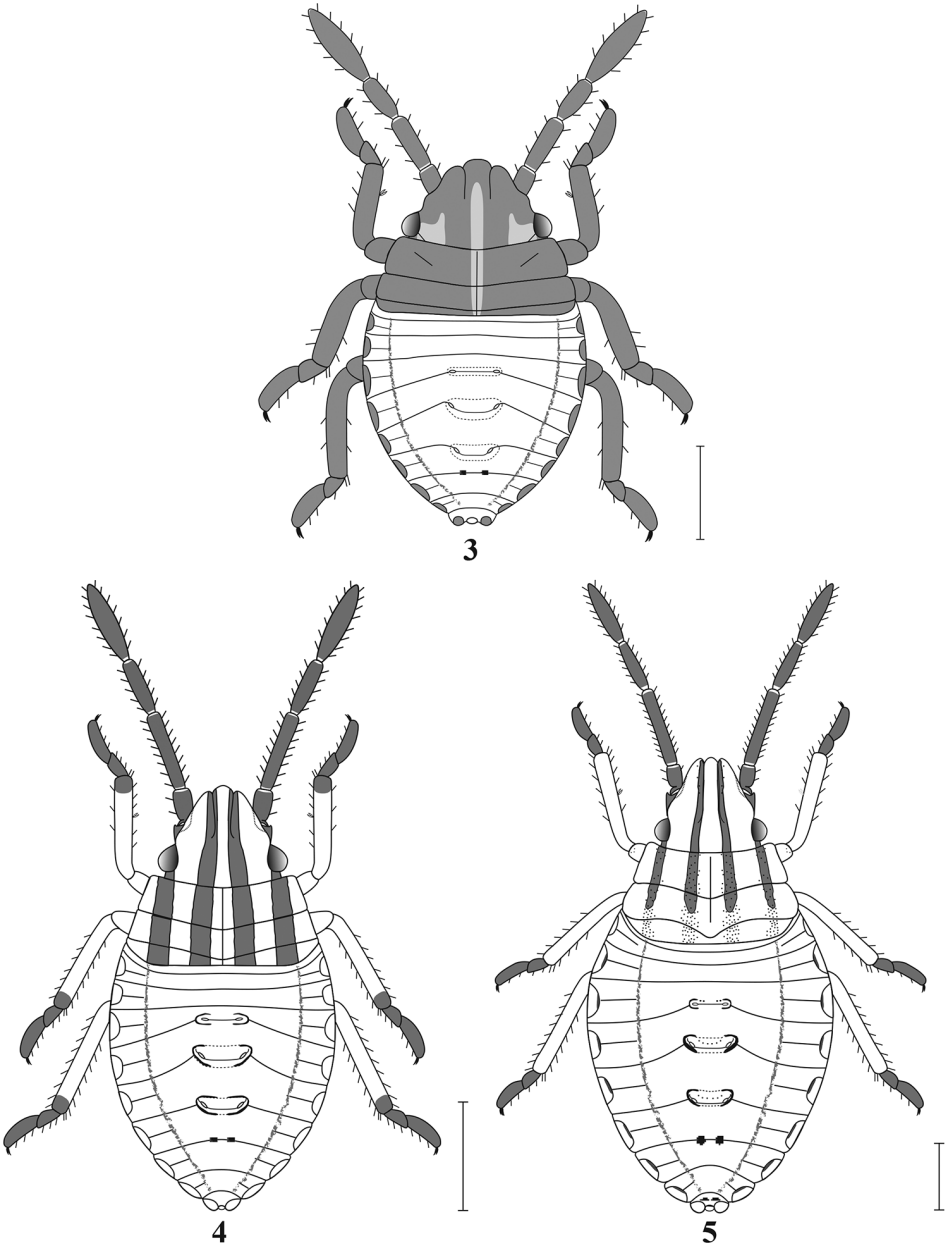
**3** First instar of *M.major* (dorsal view) **4** Second instar of *M.major* (dorsal view) **5** Third instar of *M.major* (dorsal view). Scale bars: 0.5 mm.

***First Instar* (Fig. 3).** Length, 1.18 ± 0.03 (1.16 ± 0.03); width, 0.72 ± 0.03 (0.68 ± 0.02). Body elliptical, becoming more elongate during stadium; widest at abdominal segments 2–4; yellowish brown, or yellowish brown with head and thorax brown.

Head yellowish brown to light brown, often with two broad, dark brown, longitudinal stripes submedially, running from apex of tylus to near or reaching posterior margin of head; yellowish-brown region mesad of eye, sometimes extending posteromedially to posterior margin of head and merging medially with that of other side; head declivent, anterolateral margins sinuate; tylus distinctly exceeding juga in length. Antennae 4-segmented, often concolorous with head; dorsal surface of segment 2 with dorsomedial surface not carinate, rounded in cross section; apical segment longest, fusiform; ratio of antennal segment lengths ≈1:1.8:1.4:2.8. Labium 4-segmented, generally concolorous with head, segment 4 often darker, particularly distally.

Thoracic nota yellowish brown to darker laterally; faint yellowish medial stripe present; dark brown longitudinal stripes absent. Pro- and mesonota completely sclerotized, medial areas weakly extended posteriorly, posterior margins straight either side of midline. Metanotum sclerotized only in anterior 1/2, forming plate, this plate narrowed medially, posterior margin straight; posterior margin of segment (i.e., membranous area) also straight; mediolongitudinal line extending from anterior margin of pronotum to posterior margin of metanotal plate. Ratio of pro-, meso-, and metanota (sclerotized and membranous portions combined) ≈1:0.7:0.3. Pleura and sterna brown to yellowish brown. Legs concolorous with body except for distal tip of tarsomere 2, which may be dark brown.

Abdomen yellowish brown dorsally with thin brownish sublateral longitudinal stripe either side and, often, with reddish markings. Faint brown medial (3–4) and lateral (9) plates present: medial plates poorly defined; plate 1 obscure, narrow, rectangular, plates 2–3 subquadrate, plate 4, when present, minute, paired, oval; plates 1–3 each with paired ostioles; lateral plates extending dorsally and ventrally from lateral edge of abdomen: plate 1 rounded dorsally; plates 2 – 9 subquadrate; faint pseudointersegmental lines present mesad of lateral plates 1–8. Ventral surface mostly concolorous with corresponding dorsal surface, ventral extensions of lateral plates similar to dorsal extensions. Abdominal spiracles on segments 2–8, each near lateral margin of corresponding segment. Single trichobothrium (primary trichobothrium) posteromesad of each spiracle on segments 3–7, arising from dark brown sclerite.

***Second Instar* (Fig. 4).** Length, 2.06 ± 0.03 (2.02 ± 0.03); width, 1.09 ± 0.02 (0.92 ± 0.02). Body more elongate.

Head, dorsally, with pair of distinct dark submedial stripes, extending from inner margin of juga (and outer margin of tylus) to posterior margin of metanotum; short lateral dark stripe extending from near antenniferous tubercle through eye to posterior margin of head, anterolateral margins straight to weakly sinuate (depending upon angle of view); tylus distinctly longer than juga. Dorsal surface of antennal segment 2 with dorsomedial surface not carinate, rounded in cross section; ratio of antennal segment lengths ≈1:2.6:1.9:2.6.

Thorax, dorsally, with pair of distinct sublateral dark brown stripes in addition to submedial pair. Pro- and mesonota with medial areas moderately extended posteriorly, posterior margins straight either side of midline. Ratio of pro-, meso-, and metanota ≈1:0.7:0.2. Pleural area light brown with one to two longitudinal stripes, one dorsopleural, the other ventropleural; dorsopleural stripe always present, extending from posterior margin of head to posterior margin of thorax, a much narrower ventropleural stripe occasionally present (absent in lighter individuals) adjacent to coxae, paralleling dorsopleural stripe. Tarsi dark brown, often extending to distal portion of femur in dark individuals.

Abdomen, dorsally, with medial plates more heavily sclerotized; plates 1–3 with lateral/sublateral margins dark brown extending mediad, in darker specimens, extensions of plates 2–3 may reach almost to the midline; plate 4 present, slightly larger. Ventrally, sclerite surrounding each primary trichobothrium larger. Second, smaller trichobothrium (secondary trichobothrium) present on segments 3–7, adjacent to and slightly laterad of primary trichobothrium, each arising from dark brown sclerite, sclerite smaller than that associated with primary trichobothrium. Otherwise, like first instar.

***Third Instar* (Fig. 5).** Length, 3.17 ± 0.07 (3.16 ± 0.06); width, 1.75 ± 0.04 (1.31 ± 0.01). Head with juga and tylus subequal in length. Dorsal surface of antennal segment 2 with dorsomedial surface carinate, segment subtriangular in cross section, slightly widening basally; ratio of antennal segment lengths ≈1:2.7:1.8:2.1.

Mesonotum with medial area strongly extended posteriorly, posterior margin rounded medially, weakly arcuate laterally; mediolongitudinal line faint, usually extending from anterior margin of pronotum to near posterior margin of mesonotum. Submedial and sublateral stripes less distinct, often broken or reduced to a series of punctures, particularly caudad. Ratio of pro-, meso-, and metanota ≈1:0.9:0.1. Pleura with ventropleural stripe absent, region concolorous with thorax.

Abdomen with lateral plates, dorsally and ventrally, often darkly pigmented along lateral margins. Ventrally, sclerites surrounding primary trichobothria larger, often evident as distinct spots in darker specimens. Otherwise, like second instar.

**Figures 6–9.**
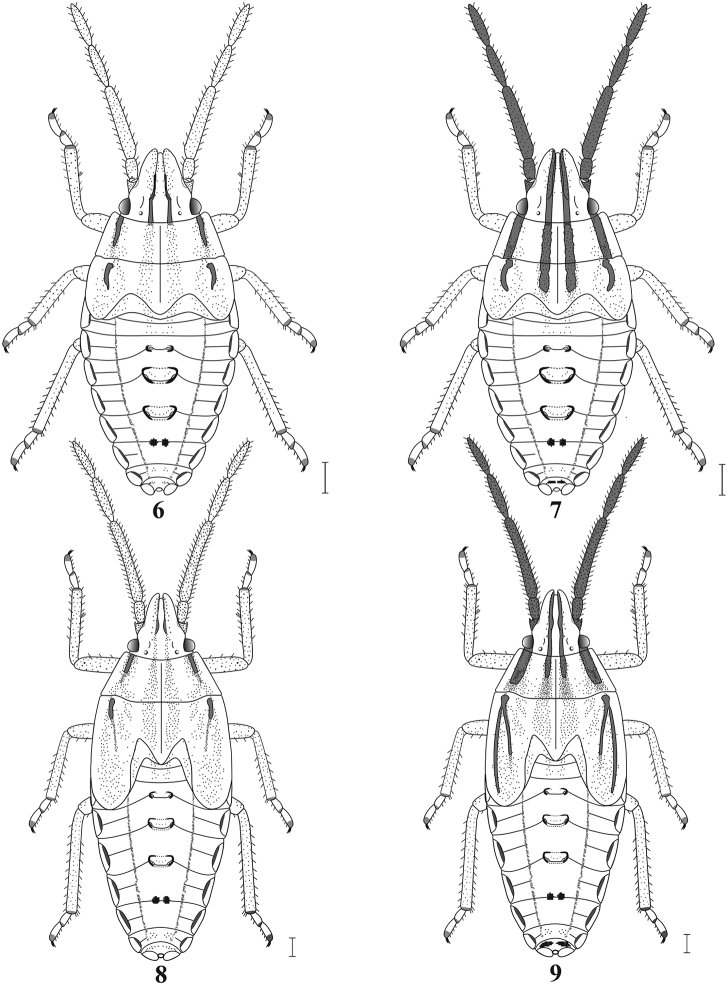
**6** Fourth instar of *M.major*, light form (dorsal view) **7** Fourth instar of *M.major*, dark form (dorsal view) **8** Fifth instar of *M.major*, light form (dorsal view) **9** Fifth instar of *M.major*, dark form (dorsal view) Scale bars: 0.5 mm.

***Fourth Instar* (Figs 6, 7**). Length, 4.51 ± 0.15 (4.53 ± 0.15); width, 2.40 ± 0.09 (2.00 ± 0.04). Two color forms present, one light , one dark. Head with juga exceeding tylus in length. Ocelli visible between compound eyes. Dorsally, submedial brown stripes of head, nota, and sublateral stripes of nota further reduced to punctures; dark punctures generally more diffuse, particularly on wing pads. Antennal segments 1–4 with brown punctures, ground color of lighter specimens concolorous with head; antennal segment 2 more widening basally; ratio of antennal segments ≈1:3.2:2:2. Mesonotum with posterior margin rounded medially, strongly arcuate laterally; wing pads evident, extending to abdominal segment 2. Metanotal wing pads evident but not as well defined as anterior pair. Ratio of pro-, meso-, and metanota ≈1:1.2:0.1. Legs generally concolorous with thorax, dark areas often reduced to tips of tarsomeres. Pleura with dorsopleural stripe present as a curved brown line laterally per segment, each enclosing a series of brown spots. Abdomen, dorsally, often with an irregular series of transverse spots submedially on segments 8–9, particularly in dark forms. Ventrally, sclerite surrounding each primary trichobothrium larger, usually evident as distinct spot; dark forms often with dark brown median longitudinal spots on one or more segments. Otherwise, like third instar.

***Fifth Instar* (Figs 8, 9**). Length, 7.62 ± 0.17 (7.78 ± 0.17); width, 2.99 ± 0.11 (2.92 ± 0.06). Two color forms present, one light, one dark. Body widest at mesonotum. Antennal segments more heavily punctate; ratio of antennal segments ≈1:3.7:2.1:1.8. Thorax, dorsally, with punctures more numerous and diffuse; wing pads well developed, mesonotal pads extending to abdominal segments 3–4, metanotal pads ≈same length. Ratio of pro-, meso-, and metanota ≈1:1.4:0.04. Otherwise, like fourth instar.

## Diagnosis

The five instars are readily distinguishable by characters other than differences in body size. The first instar differs from later instars by the absence of distinct dorsal submedial and sublateral longitudinal stripes on the nota; the thoracic pleura, which are completely brown and lack stripes; and the presence of a single trichobothrium posteromesad of each spiracle on segments 3–7. Older instars have distinct submedial and sublateral stripes on the nota, the thoracic pleura usually have two longitudinal stripes, one dorsal, one ventral; and two trichobothria are present posteromesad of each spiracle on abdominal segments 3–7. The second instar can be distinguished from older instars by the juga, which exceeds the length of the tylus; the dorsal surface of antennal segment 2, which is rounded; the posterior margin of the mesonotum, which is moderately extended medially, straight either side of midline; and the presence of two well-developed thoracic pleural stripes, which are unbroken. Older instars have the juga equal to or longer than the tylus; the dorsal surface of antennal segment 2 is carinate; the posterior margin of the mesonotum is strongly extended medially, arcuate laterally; and the thoracic pleural stripes are broken. The third instar can be distinguished from the fourth and fifth instars by the juga and tylus, which are equal in length, posterior margin of mesonotum, which is weakly arcuate laterally, and by the lack of wing pads. The fourth and fifth instars have the juga distinctly longer than the tylus, posterior margin of the mesonotum is strongly arcuate laterally, and the wing pads that are easily discernible. The fourth and fifth instars can be distinguished by the lengths of the wing pads, which reach abdominal segment 2 in the fourth and abdominal segments 3 or 4 in the fifth.
